# Supplementary material for: Enhanced visible-light photocatalytic activity of anatase-rutile mixed-phase nano-size powder given by high-temperature heat treatment
Source: R Soc Open Sci. 2020 Jan 15;7(1):191539. doi: 10.1098/rsos.191539 (PMC7029928; doi:10.1098/rsos.191539)
Supplement: Supporting Information [file rsos18539supp1.docx]

Supporting Information

Enhanced visible light photocatalytic activity of

anatase-rutile mixed-phase nano-size powder given by

high-temperature heat treatment

Takamasa Ishigaki*^1,2,3^, Yusuke Nakada^2^, Naoki Tarutani^†1^,

Tetsuo Uchikoshi^3,4^, Yoshihiro Tsujimoto^4^, Masaaki Isobe^4^,

Hironori Ogata^1,2,3^, Chenning Zhang^4^ and Dong Hao^3^

1 Department of Chemical Science and Technology, Hosei University, 3-5-4 Kajino-cho, Koganei, Tokyo 184-8584, Japan;

2 Department of Applied Chemistry, Graduate School of Science and Engineering, Hosei University, 3-5-4 Kajino-cho, Koganei, Tokyo 184-8584, Japan;

3 Research Center for Micro-Nano Technology, Hosei University, 3-11-15 Midori-cho, Koganei, Tokyo 184-0003, Japan;

4 Research Center for Functional Materials, National Institute for Materials Science, 1-2-1 Sengen, Tsukuba, Ibaraki 305-0047, Japan.

* Author for correspondence ([ishigaki@hosei.ac.jp](mailto:ishigaki@hosei.ac.jp)).

† Present address: Department of Applied Chemistry, Graduate School of Engineering, Hiroshima University, 1-4-1, Kagamiyama, Higashi-Hiroshima, Japan, 739-8527, Japan

1. Near intrinsic characteristic of P25 TiO_2_ powder

Near intrinsic characteristics of P25 TiO_2_ powder were examined by measurements of impurity concentration and magnetic properties. For comparison, the measurement was made on a plasma-synthesized non-doped TiO_2_ nano-size powder [1]. The plasma-synthesized TiO_2_ powder was prepared by oxidizing the mist precursor solution with an Ar/O_2_ radio-frequency (RF) thermal plasma. A liquid precursor, Titanium n-butoxide [Ti(OC_4_H_9_)_4_], was used as titanium (IV) source. Diethanolamine [DEA, HN(OC_2_H_5_)_2_] was mixed (molar ratio: Ti(OC_4_H_9_)_4_:DEA = 1:4) to prevent hydrolysis of the source alkoxide by water or moist air. The liquid precursor was injected into the plasma reactor as a mist.

Both P25 and plasma-synthesized powders were dissolved in hot mixed aqueous solution of hydrochloric and hydrofluoric acids, and were analyzed using an inductively coupled plasma-optical emission spectrometer (ICP-OES) (model ICPS-8100, Shimadzu Corp., Kyoto, Japan). The elemental concentrations of impurity transition metal elements, Fe, Co, Ni and Cr, were measured. Magnetic properties were measured at room temperature and from 10-350 K using a SQUID instrument (Model MPMS-XL, Quantum Design, San Diego, CA, USA).

Measured elemental concentrations of impurity transition metal elements were given in Table S1. Although the impurity concentrations in the two powders are similarly very low, the P25 powder is almost white and the plasma-synthesized powder has a color of very pale beige. The coloration of plasma-synthesized TiO_2_ powder, even if it is quite pale, gives us the idea of formation of Ti^3+^ and oxygen vacancies in the plasma-synthesized TiO_2_ powder, which was prepared through a rapid cooling process in the thermal plasma processing.

Formation of Ti^3+^ and oxygen vacancies in TiO_2_ may be formed through a defect reaction:

TiO_2_ ⇆ Ti_Ti_’ + O_O_^x^ + V_O_^··^ + e’ + 1/2O_2_ (1)

where Ti_Ti_, O_O_, V_O,_ and e denote the titanium ions at titanium sites, oxygen ions at oxygen sites, vacancies at oxygen site and electrons, respectively, and symbols, ^•^, ^x^ and ’ are the Kröger-Vink notations for the net charge +1, the zero net charge, and the net charge -1, respectively [2]. For the impurity ions of lower valence than Ti^4+^ ions, for example, the dissolution of Fe^3+^ in TiO_2_ can be expressed by the equation,

Fe_2_O_3_ ⇆ 2Fe_Ti_’ + 3O_O_^x^ + V_O_^··^ (2)

where Fe_Ti_ denotes the iron ions at titanium sites. A certain amount of dissolution of Fe^3+^ leads to the advance of the defect reaction (2), and the reaction (1) tends to proceed to the left-hand side. That is, the defect formation is almost controlled by the Fe dissolution, and the Ti^3+^ concentration is considerably decreased.

Results of magnetization measurement for the two powders are shown in Figure S1. The plots were thus obtained. The M-H data was corrected by subtracting values for the sample capsule of diamagnetic property.

It can be evaluated that the Ti^3+^ concentration in the plasma-synthesized non-doped TiO_2_ powder is approximately 0.001, and the approximate ratio of Ti^3+^, which contributes to the ferromagnetic property, is 0.1 of total Ti^3+^. The MH curve of the plasma-synthesized non-doped TiO_2_ powder shows the weak ferromagnetic property (Fig. S1(a)). In the right-hand Y-axis, the conversion values in the mμ_B_ unit are shown. The value of saturation magnetization, ~6x10^-6^_,_ is ~1x10^-5^ of 1 μ_B_ (=5585 emu/mol), which is the value for the 1 mole of Ti^3+^ spins with total spin angular momentum, S=1/2. This means the concentration of Ti^3+^ spins, which contributes to the ferromagnetic property, is ~10 ppm in TiO_2_. The temperature dependence of M/H in Fig. S1(b) gives the evaluated value of Curie constant, 3.33x10^-4^ by a least-squares fitting. The value is ~1x10^-3^ of 0.375, which is the theoretical value of Curie constant for the 1 mole of Ti^3+^ with the total spin angular momentum, S=1/2. It can be evaluated that the concentration of Ti^3+^ is ~0. 1mol% in TiO_2_, assuming all Ti^3+^ contribute to the paramagnetic property.

In contrast, the M-H curve of commercial P25 powder in Fig. R1(a) does not show obviously the ferromagnetic property, but the paramagnetic property, as the plots are much scattered. As the raw data was of diamagnetic property, the M-H data was corrected by subtracting values for the sample capsule of diamagnetic property. The magnetization of P25 as shown in the figures is recognized to correspond to the contribution of the impurity magnetic metal elements with very low concentration. It is also concluded that, in as-received P25 powders, concentration of Ti^3+^ ions should be quite low.

Table S1 Concentrations of impurity transition metal elements in P25 and a plasma-synthesized non-doped TiO_2_ nano-size powders[1].

| Sample | Concentration, mass% | | | |
| --- | --- | --- | --- | --- |
|  | Fe | Co | Ni | Cr |
| P25 | 0.013 | 0.009 | 0.010 | <0.001 |
| Non-doped TiO_2_ | 0.013 | 0.009 | <0.001 | <0.001 |

**(a)**

**(b)**

Figure S1 (a) Magnetization properties at 300 K and (b) the temperature dependence of M/H of the P25 and a plasma-synthesized non-doped TiO_2_ nano-size powders.

1. Evaluation procedure of Cl concentration in powders using thermal desorption spectroscopy [3].

Chlorine concentration was determined semi-quantitatively. The species adsorbed and chemically bonded chlorine in the powder were analyzed using thermal desorption spectroscopy (TDS: ESCO EMD-WA1000, Musahino, Tokyo, Japan), during which the powder specimens were heated under a high vacuum condition of ~10^-8^ Pa in the temperature range of 300-1000°C. The gases evolved were detected by a quadrupole mass spectrometer (QMS: Pfeiffer Vacuum Technology AG, QMG421, Asslar, Germany). Desorption peaks at low temperatures <300°C were due to the presence of physically bonded surface chlorine, whereas those at high temperatures >300°C from chemically bonded ones.

Correspondence between the QMS signal intensity and the gas concentration was evaluated using the equation

N_X_= A_X_ · (N_S_ /A_S_) · (FF_X_ · XF_X_ · TF_m_ · Q_m_)_s_ / ( FF_xm_ · XF_x_ · TF_m_ · Q_m_)_x_,

where N is the desorbed number, A is the peak area of the molecule, FF is the fragmentation factor, XF is the ionization factor, TF_m_ is the transmission factor of mass number M relative to 28, Q_m_ is the pumping speed of the turbo molecular pump at mass number M, and the subscripts S and X denote the values for the standard sample (H_2_) and species X, respectively. As the QMS transmission factor differs from the theoretical value, 28/MW_m_, and each QMS has different values, we corrected the transmission factor by calibration using H-implanted (1×10^16^/cm^2^) and Ar-implanted (5×10^15^/cm^2^) Si wafers. The values for FF, TF, and Q were taken from the technical report of the QMG 421 quadrupole mass spectrometer. The signal intensity ratio of Cl to H was thus converted to desorbed number ratio.

3. Specific surface area and pore size distribution.

Porous characteristics of samples were analyzed by N_2_ sorption measurements (Belsorp-18 II, MicrotracBEL Corp., Osaka, Japan). Prior to N_2_ sorption measurements, samples were outgassed under vacuum at 200°C for 6 h.

Physisoption isotherms are shown in Fig. S2 (a). Isotherms measured with three powders, as-received P25 powder and powders heat-treated at 700 and 800^o^C, were of type II, while only that of as-received P25 powder shows the type H1 hysteresis loop [4].

The specific surface area was estimated according to the Brunauer–Emmett–Teller method, and the pore volume distribution was calculated using the Barrett–Joyner–Halenda method, which is given in Fig. S2 (b). Evaluated values of specific surface area and pore volume are listed in Table S1. Evaluated values and distribution of heat-treated powders would be much less accurate due to much smaller surface area and pore volume.

Table S2 Evaluated values of specific surface area and pore volume for as-received P25 powders and powders heat-treated at 700 and 800^o^C.

|  | Specific surface area  / m^2^ g^−1^ | Pore volume  / cm^3^ g^−1^ |
| --- | --- | --- |
| As-received P25 powder | 44.0 | 0.228 |
| Powder heat-treated at 700^o^C | 2.7 | 0.083 |
| Powder heat-treated at 800^o^C | 1.7 | 0.068 |





Figure S2 (a) Measured sorption isotherms and (b) evaluated pore volume distribution of as-received P25 powders and powders heat-treated at 700 and 800^o^C.

4. Survey scan XPS spectra.

X-ray photoelectron Spectroscopy (XPS) was performed by an X-ray photoelectron spectrometer (Phi-5600, Physical Electronics, Inc., Minnesota, USA) using monochromatized Al K*α* at *hv* = 1486.6 eV. The sample powders were mounted on polished indium plate and pressed without adhesives. Survey spectra were collected with a step of 0.125 eV.





Figure S3. Survey scan XPS of as-received P25 powder and powders heat-treated at 700 and 800^o^C.

References

1. Zhang CN, Ikeda M, Isobe M, Uchikoshi T, Li JG, Watanabe T, Ishigaki T. 2011. Phase composition and magnetic properties of niobium–iron codoped TiO_2_ nanoparticles synthesized in Ar/O_2_ radio-frequency thermal plasma. *J. Solid State Chem.* **184**, 2525-2532. (doi: [10.1016/j.jssc.2011.07.025](https://doi.org/10.1016/j.jssc.2011.07.025))
2. Kroger FA, Vink HJ. 1956. Relations between the concentrations of imperfections in crystalline solids. *Solid State Phys.* **3**, 307–435.
3. [Li](https://pubs.acs.org/author/Li%2C+Ji-Guang) JG, [Ikeda](https://pubs.acs.org/author/Ikeda%2C+Masashi) M, [Tang](https://pubs.acs.org/author/Tang%2C+Chengchun) C, [Moriyoshi](https://pubs.acs.org/author/Moriyoshi%2C+Yusuke) Y, [Hamanaka](https://pubs.acs.org/author/Hamanaka%2C+Hiromi) H, Ishigaki T. 2007. Chlorinated Nanocrystalline TiO_2_ Powders via One-Step Ar/O_2_ Radio Frequency Thermal Plasma Oxidizing Mists of TiCl_3_ Solution:  Phase Structure and Photocatalytic Performance. *J. Phys. Chem. C* **111***,* 18018-18024. (doi: 10.1021/jp077320q)
4. Sing KSW, Everett DH, Haul RAW, Moscou L, Pierotti RA, Rouquerol J, Siemieniewska T. 1984. Reporting physisorption data for gas/solid systems with special reference to the determination of surface area and porosity. *Pure Appl. Chem.* **57**, 603-619. (doi: 10.1351/pac198557040603)
